# Diagnosis of Feline Infectious Peritonitis: A Review of the Current Literature

**DOI:** 10.3390/v11111068

**Published:** 2019-11-15

**Authors:** Sandra Felten, Katrin Hartmann

**Affiliations:** Clinic of Small Animal Medicine, Center for Clinical Veterinary Medicine, Ludwig-Maximilians-Universitaet Munich, Veterinaerstr. 13, 80539 Munich, Germany; hartmann@medizinische-kleintierklinik.de

**Keywords:** diagnosis, FIP, antibody, RT-PCR, immunohistochemistry, IHC, immunocytochemistry, ICC

## Abstract

Feline infectious peritonitis (FIP) is a fatal disease that poses several challenges for veterinarians: clinical signs and laboratory changes are non-specific, and there are two pathotypes of the etiologic agent feline coronavirus (FCoV), sometimes referred to as feline enteric coronavirus (FECV) and feline infectious peritonitis virus (FIPV) that vary fundamentally in their virulence, but are indistinguishable by a number of diagnostic methods. This review focuses on all important steps every veterinary practitioner has to deal with and new diagnostic tests that can be considered when encountering a cat with suspected FIP with the aim to establish a definitive diagnosis. It gives an overview on all available direct and indirect diagnostic tests and their sensitivity and specificity reported in the literature in different sample material. By providing summarized data for sensitivity and specificity of each diagnostic test and each sample material, which can easily be accessed in tables, this review can help to facilitate the interpretation of different diagnostic tests and raise awareness of their advantages and limitations. Additionally, diagnostic trees depict recommended diagnostic steps that should be performed in cats suspected of having FIP based on their clinical signs or clinicopathologic abnormalities. These steps can easily be followed in clinical practice.

## 1. Introduction

Feline infectious peritonitis (FIP) is a fatal disease that occurs in domestic and wild felids worldwide. The etiologic agent, the feline coronavirus (FCoV), occurs in two distinct pathotypes that can be distinguished by their biological behavior, but not by their morphology. Some authors use different names for these two pathotypes, although they both belong to the same virus species. The feline enteric coronavirus (FECV) is highly prevalent in multi-cat environments and highly contagious—nearly 100% of cats that get in contact with FECV from feces of shedding cats become infected. However, infection is mostly asymptomatic or only causes mild and transient diarrhea [1,2,3]. The feline infectious peritonitis virus (FIPV), in contrast, is not infectious via the fecal-oral route, but arises by mutation from the avirulent FECV within a small percentage of infected cats and then causes the fatal disease feline infectious peritonitis (FIP) [4,5,6]. It is still unknown which exact genes harbor the mutation(s) leading to FIPV development. Since promising results using new drugs for treating cats with FIP have been published recently, definitive ante mortem diagnosis is crucial in order to correctly identify the population of cats which could benefit from such antiviral treatment. At the same time, definitive diagnosis is challenging, since most existing diagnostic tests cannot differentiate between FECV and FIPV, and especially in cats without body cavity effusions, it is often difficult to reach a definitive diagnosis ante mortem.

## 2. General Laboratory Testing

FIP occurs most often in young cats under two years of age [7]. Male cats [7] and certain breeds are suggested to be overrepresented [8,9,10]. Clinical signs, such as anorexia, lethargy, weight loss, pyrexia, ocular and neurological signs like gait abnormalities or abnormal mentation, are non-specific [7,11,12,13,14]. The same is true for clinicopathologic abnormalities. In general, tests using effusion have much better predictive values than tests using blood [15,16]. Thus, ante mortem diagnosis of FIP is especially difficult in cats without significant effusion. Since a definitive diagnosis cannot be made based on signalment, history and clinical and laboratory signs alone, these parameters should always be evaluated as an entity and potentially in conjunction with other parameters, such as results of molecular or even more invasive diagnostic tests. In order to avoid falsely diagnosing FIP in unaffected cats, specificity is always the most important diagnostic value to consider.

### 2.1. Blood

Hematological abnormalities are very common in cats with FIP. Reported changes mainly include anemia (non-regenerative as well as regenerative anemia can be present, e.g., immune-mediated hemolytic anemia has been reported), microcytosis with or without anemia, lymphopenia (more commonly in cats with effusion), band neutrophilia (with or without segmented neutrophilia) and thrombocytopenia. Whereas microcytosis and band neutrophilia are common features of FIP in general, lymphopenia, observed in about 50% of cats with FIP, can be seen significantly more commonly in cats with effusion, but is reported only rarely in cats without effusion. The vast majority of cats with FIP also have abnormalities upon serum biochemistry. In particular, hyperproteinemia, and especially hyperglobulinemia (that can also be present without an increase in total serum protein), hypoalbuminemia (more common in cats with effusion), hyperbilirubinemia (more common in cats with effusion) and potentially, depending on organ involvement, azotemia (detected more often in cats without effusion) or increased liver enzyme activities can be present. The most common abnormality reported is hyperglobulinemia, documented in about 89% of cats with FIP [7]. These changes can occur in variable combinations but it is important to note that they absolutely are neither pathognomonic nor specific for FIP, meaning that they can occur in any cat suffering from various diseases with an inflammatory basis, which represent differential diagnoses for FIP [7,17,18,19,20,21,22]. Hyperglobulinemia in cats with FIP can occur either alone or in combination with hypoalbuminemia or hyperproteinemia. Although it has been suggested that the albumin to globulin (A:G) ratio has better diagnostic utility than the gamma-globulin or total protein concentration alone [16] and several cut-offs have been suggested to potentially rule in (<0.4) or rule out (>0.6–0.8) FIP [16,19,21,22,23], the A:G ratio, as other hematological and serum biochemistry changes, should only be interpreted in conjunction with signalment, history, other laboratory parameters and possibly molecular diagnostic methods. Elevated gamma-globulin concentrations can be either polyclonal (more common) or monoclonal as differentiated by serum protein electrophoresis [24]. Thus, if a cat presents with any of the aforementioned clinicopathologic abnormalities in routine practice, these always have to be interpreted in conjunction with other clinical, laboratory or imaging findings. They might only support a suspicion of FIP if other abnormalities consistent with FIP are present as well and FIP should never be diagnosed solely on the basis of hematological or biochemical parameters.

Cats with FIP often exhibit markedly increased concentrations of acute phase proteins (APP), such as alpha-1-acid glycoprotein (AGP), serum amyloid A (SAA) or haptoglobin in serum [11,25,26,27,28]. Still, although these parameters can help in establishing a diagnosis and a negative AGP measurement can potentially exclude FIP [15], on their own, they are not pathognomonic for FIP. Increased concentrations of APP can also be found in cats with various other inflammatory conditions, neoplasia or even in healthy FECV-infected cats [25,26,27,29,30,31,32]. As was stated before for hematological and serum biochemical parameters, measurement of APP, such as AGP, can only be helpful when interpreted together with other abnormalities in order to provide an index of suspicion of FIP in an individual cat [28].

### 2.2. Effusion

The majority of cats with FIP present with effusion—specifically, pleural effusion, ascites or a combination of both [7,13]. Even cases of pericardial effusion have been reported [33]. The typical fluid from a cat with FIP is viscous, straw-colored, clear to moderately cloudy and usually forms clots or strings because of its high protein content [34]. Macroscopic and cytologic examination of those effusions and determination of cell count and biochemical parameters is important in order to exclude or confirm other differential diagnoses (such as lymphoma by cytology or bacterial peritonitis or pleuritis by cytology and bacterial culture); but again, changes are not specific for FIP [11,15,35,36,37]. Although typical effusions from cats with FIP have a high protein content, low A:G ratio [16,38] and rather low total cell count [34], these findings can also occur in effusions of other etiologies. Nevertheless, in a cat which presents with typical signalment, clinical and routine laboratory abnormalities (such as for example a young cat with fever, icterus, anemia, hyperglobulinemia) and effusion, effusion analysis including cytology and bacterial culture, although not completely specific for FIP, can exclude other conditions that could confound the diagnosis and can at least help in increasing the pre-test probability for FIP.

The Rivalta’s test is a cheap and quick test that can easily be performed on effusions in clinical practice, which is why, in the authors’ opinion, it should be included in the diagnostic protocol in every cat with effusion. It has good sensitivity for excluding FIP (91–100%), meaning that whenever it is negative, other potential causes for effusion are much more likely than FIP. Therefore, especially if financial concerns limit the number of diagnostic tests that can be performed, the Rivalta’s test can at least be used to exclude FIP with satisfying negative predictive value with the advantage of no need for any automated analysis. However, its specificity was reported to be only 66–81% [16,39]. Since the Rivalta’s test is positive most likely secondary to increased amounts of protein and inflammatory mediators in a fluid, it was originally used to differentiate transudates and exudates [40], and therefore can also be positive in effusions caused by bacterial peritonitis/pleuritis or lymphoma [16]. These conditions, however, can usually easily be diagnosed by routine cytology and culture of the effusion, which should also always be performed when analyzing an effusion sample.

Another test that relies on the abundance of proteins in effusions from cats with FIP is the measurement of the delta total nucleated cell count (∆TNC) in an automated hematologic analyzer (Sysmex XT-2000iV, Sysmex Europe, Norderstedt, Denmark), measuring leucocytes in two different channels. The ratio between both channels, the ∆TNC, is higher in cats with FIP than in healthy cats, and its measurement showed quite good diagnostic accuracy. Using a cut-off of 1.7 for ∆TNC, diagnostic sensitivity of the method was 79–90%; specificity was 94–100%. Higher cut-offs of 2.5 or 3.4 even increased specificity to 100% [15,41,42].

### 2.3. Cerebrospinal Fluid (CSF)

Neurological signs are more likely to occur in the population of cats with FIP without significant effusion [7]. The CSF is often sampled after diagnostic imaging of the central nervous system in order to reach a diagnosis. Upon clinicopathological examination, an increased protein content and pleocytosis can be noted in the CSF of cats with FIP [14,43,44,45,46]. CSF cytology often reveals a mixed or suppurative inflammation; mononuclear infiltration can be seen as well. Unremarkable CSF cytology can also occur [46]. However, all these changes can also be present in cats with other neurologic diseases, and some cats with neurological signs caused by FIP have normal CSF analysis [45].

### 2.4. Aqueous Humor

Eye involvement, which includes pyogranulomatous and granulomatous uveitis and chorioretinitis with a mixed uveal inflammatory cell infiltrate and predominance of B lymphocytes and plasma cells [47,48,49], was reported in up to 29% of cats with confirmed FIP [13]. Aqueocentesis can provide aqueous humor for cytological and clinicopathological testing; however, cytology is often non-diagnostic or more useful to diagnose lymphoma [50,51,52].

## 3. Detection of Anti-FCoV Antibodies

FCoV infection leads to the production of specific antibodies, independently of the development of FIP [53,54,55]. After experimental infection, serum antibodies are detectable as early as seven to 28 days after infection [3,6,54,56,57,58,59].

The first assay to detect anti-FCoV antibodies was developed in 1976 [60]. By now, there are a number of different test methods: indirect immunofluorescence antibody tests (IFAT) using either FCoV [60,61,62] or transmissible gastroenteritis virus (TGEV) [62,63,64,65] as antigen, virus neutralization (VN) assays, enzyme-linked immunosorbent assays (ELISA) [62,66,67] and rapid immunochromatographic tests (RIM) [62]. An ideal test should work on different fluids, such as serum and effusion, have high sensitivity and specificity, should allow the use of small sample quantities, provide a quantitative value and a rapid result. For excluding a diagnosis of FIP (if at all possible by antibody detection), all of these aspects matter. For FCoV screening, especially sensitivity and test rapidity are important [62].

### 3.1. Blood

In contrast to what was believed earlier, it is now well-accepted that antibody tests cannot differentiate between antibodies against FECV and FIPV, and therefore, even high antibody titers in blood are not a specific indicator for FIP [6,68]. Additionally, there is evidence of cross-reactivity between FCoV and other coronaviruses, such as TGEV and canine coronavirus (CCV) [68,69]. A large proportion of the cat population (up to 80% and more, especially in multi-cat households) has serum antibodies against FCoV, but most of these cats never develop FIP [54,60,70,71,72,73,74]. Moreover, antibodies can be detected in the serum of cats that were vaccinated against FCoV [75,76,77]. The significance of the presence of antibodies for diagnosing FIP in an individual cat therefore is very limited [11,16,19].

Opinions on the value of antibody measurement for the diagnosis of FIP vary, but based on the results of numerous studies, which are shown in the following paragraphs, it is the authors’ opinion that antibody measurement is of no use in a cat suspected of having FIP. If antibodies are measured in a cat suspected of having FIP, then a titer should be determined in any case. Especially low and medium titers are of zero diagnostic value for the diagnosis of FIP [16,78]. Although a rising titer can sometimes be detected during progression of the disease [55,60], this can also be seen in conjunction with FECV reinfection, and thus is not an indicator for FIP [70]. High and rising titers can also be found in healthy FECV-infected cats [70,73] and should never be used to confirm a suspicion of FIP. Likewise, a negative antibody test result cannot exclude FIP [79,80]. In end-stage FIP, especially with fulminant effusions, declining antibody titers are possible and sometimes antibody concentrations can even drop below the limit of detection [60,68]. Approximately 10% of cats with FIP do not have serum antibodies [16]. Most likely, the immune-mediated vasculitis in these cats leads to extravasation of blood components including antibodies into the effusion, and as a consequence, they can no longer be detected in the blood by antibody assays. Additionally, a large number of antibodies is most likely bound by the high virus load in end-stage FIP [81,82,83], and thus, cats with FIP without effusions can have negative serum anti-FCoV antibody titers [84]. Moreover, it has been demonstrated in longitudinal studies that in healthy cats originating from environments endemic for FIP, antibody titers can vary greatly over time [85,86]. Therefore, measurement of a single antibody titer at a single random time point does not provide any diagnostic information. The results of studies evaluating the performance of antibody tests for diagnosing FIP are summarized in Table 1. Sensitivity and specificity partially vary depending on the antibody cut-off titer used. Most studies reported the diagnostic value of antibody measurement compared to histopathology [16,19,78]. One study included cats with FIP in which the diagnosis was established upon a combination of tests performed ante mortem and histopathology [79]. Another study included cats in which FIP was suspected clinically, but the diagnosis was not confirmed [87]. The studies included control groups consisting of either healthy cats [79] and/or cats with diseases other than FIP [19,79]; in most studies, FIP was an important differential diagnosis for all control cats [16,19,78].

As stated before, the detection of antibodies in plasma or serum (no matter which titer) is not proof for the presence of FIP. In an attempt to increase specificity, a few years ago, an antibody assay was developed that only detected antibodies against the FCoV 7b protein. This test was designed based on the erroneous assumption that FIPV, but not FECV, contains an intact 7b gene. However, the test did not result in a better diagnostic performance than other antibody assays, as both cats with FIP and FECV-infected cats (healthy or suffering from other diseases) had anti-7b antibodies [79].

Nevertheless, measurement of serum antibodies is useful in guiding preventative measures and can be used for FCoV control in multi-cat households. As such, antibody detection can be performed to screen cats that are about to be newly integrated into a group, it can help to confirm successful elimination of FCoV in groups of cats and can guide separation of infected and non-infected cats [61]. Cats without detectable antibodies most likely do not shed FCoV with their feces [62,70,85]. However, fecal shedding has been observed in experimentally infected cats despite the absence of serum antibodies [54].

### 3.2. Effusion

The diagnostic utility of anti-FCoV antibody detection in effusion has been examined in a few studies as well [16,87]. In cats with FIP confirmed by histopathology, antibody detection in effusion had a sensitivity of 86% and a specificity of 85% [16]. Although these numbers sound more promising than what is reported for the antibody detection in serum, it has to be emphasized that the diagnostic value of antibody measurement in both serum and effusion is similarly low and bears the same limitations. Additionally, it has to be mentioned that many studies on antibody detection in effusion suffer from some limitations, such as inclusion of cats without a definitive diagnosis of FIP. For example, a comparison of antibody detection in serum and effusion in cats with a clinical suspicion of FIP revealed good concordance of test results in both body fluids; the antibody titer measured in effusion was lower than that determined in serum in 23% of cats. However, FIP was not definitively confirmed in any of the cats included [87]. The same was true for a study in which FIP was suspected in 61 cats based on different effusion features (cases had to fulfill all or most of the criteria for FIP diagnosis given by the European Advisory Board of Cat Diseases recommendations of 2009 [88]), but the diagnosis was not definitively confirmed in any of the cats. Detection of anti-FCoV antibodies had a sensitivity of 84%. The fact that 18 cats without detectable antibodies were positive for FCoV RNA in their effusion demonstrated that anti-FCoV antibody detection alone has only limited diagnostic value [89]. One study even found an inverse correlation between FCoV antibody titers and FCoV viral load; therefore, false negative antibody results can occur in cats with high viral RNA loads [81]. In conclusion, antibody detection in effusion is not more useful for the diagnosis of FIP than antibody detection in serum (Table 2).

### 3.3. CSF

While the detection of anti-FCoV antibodies in the CSF of cats with neurologic FIP was first regarded as useful for the diagnosis [45], subsequent investigations including larger and more appropriate control groups showed that the detection of antibodies in the CSF, as in serum and effusion, is not suitable for a definitive ante mortem diagnosis of FIP [46]. Sensitivity and specificity were calculated with histopathology as reference standard; the control groups either consisted of only cats with any kind of neurologic disease [45] or of a combination of cats with neurologic and non-neurologic disorders [46] (Table 3). A recent study included cats with neurological signs suggested to be caused by FIP, although the diagnosis was not confirmed by histopathology. In this study, CSF from a large number of cats was analyzed and revealed a low sensitivity of the detection of FCoV antibodies for the diagnosis of FIP. However, the authors hypothesized that the detection of FCoV RNA in CSF would confirm FIP and, as they found FCoV RNA in all cats with a CSF antibody titer of 1:640 or higher, based on these results concluded that a CSF antibody titer of 1:640 or higher might be confirmatory for FIP. However, the results are of limited value, as control cats were not included in the study and the diagnosis of FIP was not confirmed [90].

Due to the correlation of serum and CSF antibodies and the fact that antibody titers measured in serum were always higher than those measured in CSF, extravasation of antibodies from serum into the CSF across an impaired blood-brain barrier is discussed as the most likely explanation for the detection of anti-FCoV antibodies in the CSF of cats without FIP. Another possibility seems to be local antibody production by migrating B lymphocytes in cats with FECV infection [46], whereas there is no evidence for specific antibody production by intrathecal B lymphocytes [44].

## 4. Detection of Immune Complexes

The production of non-protective antibodies and formation of immune complexes are discussed as key features for the development of typical immune-mediated lesions in cats with FIP [56,91,92,93,94,95,96,97], and circulating immune complexes could be detected in serum and effusion of FCoV-infected cats [53,54,95,98,99,100]. Compared to histopathology as reference standard, the detection of immune complexes in serum via competitive enzyme-linked immunosorbent assay (ELISA) had a sensitivity of 48% and a specificity of 91% for diagnosing FIP (Table 4). Control cats in that study were suspected of having FIP, but another disease was diagnosed [16].

Other investigations confirmed the presence of FCoV-specific immune complexes in healthy cats without FIP [53,54]. It has been suggested that also in FECV-infected cats, immune complexes seem to circulate, although for a limited period of time only [99]. In conclusion, detection of immune complexes is not useful for the diagnosis of FIP [16].

## 5. Detection of FCoV Antigen in Macrophages by Immunostaining

Monocytes/macrophages are the target cells for FIPV replication [91,101,102,103,104,105]. Both FECV and FIPV can replicate within monocytes/macrophages and lead to monocyte-associated viremia [54,106,107,108,109]. For a long time, it was believed that only FIPV can replicate in a sufficient way that allows intracellular antigen detection via immunologic methods [88], in which antibodies bind to FCoV antigen within its target cells and enzymatic reactions or fluorescent conjugates result in coloring of the antigen. However, this assumption has been questioned recently, as a number of studies, which will be shown in the following paragraphs, have detected positive immunostaining also in cats not suffering from FIP. The nature of these false positive results remains unclear; it is possible that not only FIPV, but also FECV can be detected intracellularly in macrophages via immunologic staining methods.

### 5.1. Tissue

Immunohistochemical staining of FCoV antigen within characteristic histopathological tissue lesions is still considered gold standard for the diagnosis of FIP [11,12,13,102,110]. When first reported in 1989, a study described the detection of FCoV antigen in epithelial cells of a cat’s nictitating membrane by immunofluorescence staining of impression smears [111]. A different study used an avidin-biotin-complex (ABC) method to detect FCoV antigen in tissue samples of various organs by enzymatic color change [112]. Since then, successful immunostaining of antigen has been demonstrated several times in different organs from cats with FIP—either by indirect immunofluorescence [113,114] or by immunohistochemistry (IHC) within tissue macrophages in histopathologically evident tissue lesions caused by FIP [102,115,116]. IHC was shown to have excellent sensitivity of 97–100% in cats with histopathologically confirmed FIP [115,116] and also specificity of up to 100% to exclude FIP in control cats with other diseases diagnosed by histopathology [115]. An overview of studies reporting the detection of FCoV antigen in tissue samples is shown in Table 5.

In many cases, IHC can only be performed post mortem, because ante mortem tissue sample collection via laparotomy or laparoscopy is an invasive procedure that bears several risks for the sick cat. Many different organs have to be sampled due to the fact that FCoV antigen is variably distributed within the FIP-induced tissue lesions [102]. Unfortunately, the use of liver, kidney or lymph node samples obtained by minimally invasive ultrasound-guided fine-needle aspirates (FNA) or Tru-cut biopsies (TCB) for immunocytochemical or immunohistochemical staining has been shown to result in low sensitivities [117,118]. A possible explanation for this could be inadequate material obtained for analysis due to cellular damage [119]. Additionally, a false positive immunocytochemical result was found in a mesenteric lymph node sample, resulting in a specificity of only 91% [118]. Therefore, immunocytochemistry (ICC) or IHC on samples obtained by FNA or TCB cannot reliably confirm or exclude FIP as a sole diagnostic test [117,118].

Besides its diagnostic use, IHC has also been applied to detect FCoV antigen in macrophages in unusual tissues or non-domestic felids. As such, FCoV antigen was detected in the skin of two cats with atypical skin lesions caused by FIP [120,121], in the penile tissue of a male cat [122], in different tissues obtained from a mountain lion [123] and in ocular tissues from a lion [124].

### 5.2. Effusion

Most often, immunofluorescence staining has been performed to detect FCoV antigen in effusions. For this, fluorescein-conjugated antibodies are used to detect FCoV antigen within the cytoplasm of macrophages [125]. In earlier studies, this test was reported to have an excellent specificity of 100%. Therefore, it was believed that a positive result would in all cases allow a definitive diagnosis of FIP [16,78,125,126]. However, more recent investigations also showed positive results of immunofluorescence or ICC in cats with effusions caused by other diseases than FIP. Possible explanations for false positive results include binding of the antibody to cellular structures within macrophages (non-specific staining) or systemic spread and subsequent detection of FECV in cats without FIP [127,128].

Sensitivity of immunostaining in effusion is not absolute; thus, a negative test result cannot exclude FIP [16,78,125,126,127]. Low numbers of macrophages in effusion samples or competitive binding of FCoV antigen by circulating antibodies in the effusion are possible reasons for false negative results. One study demonstrated that all cats with false negative test results had anti-FCoV antibodies in serum or effusion [78]. Only one study reported a sensitivity of 100% for the detection of FCoV antigen in effusion by immunofluorescence staining [128]. In contrast to other studies, which examined effusion samples obtained both ante and post mortem [125,126,127], this study only included samples collected ante mortem [128]. Additionally, a monoclonal biotinylated antibody was used, whereas previous studies applied a polyclonal fluorescein-conjugated antiserum in their staining protocols [16,125,126]. Finally, samples were analyzed within 24 hours after collection [128] and longer intervals between sampling and analysis could result in decreasing sensitivities. A decreasing sensitivity could be observed with increasing time interval between sample collection and analysis, although FCoV antigen could still be detected for at least two days in samples stored at 4 °C or room temperature [128]. Table 6 shows all studies investigating the detection of FCoV antigen in effusion. Reference standard was either histopathology [16,78,125,128] and/or IHC [126,127]. Control groups consisted of cats with clinical signs consistent with FIP but a definitive diagnosis of another disease [16,78,125,126,127,128].

### 5.3. CSF

It is also possible to detect FCoV antigen in the CSF of cats with FIP (with and without neurological signs) [129,130]. The only prospective study to date reported a sensitivity of 85% and a specificity of 83% for the immunocytochemical demonstration of FCoV antigen in macrophages in the CSF of cats with and without neurological signs (Table 7). In this study, IHC was the reference standard for the diagnosis of FIP; control cats had clinical signs typical for FIP, but other diseases were diagnosed histopathologically and IHC was negative in all cats. If only cats with neurological signs were evaluated, sensitivity surprisingly decreased to 78%, whereas specificity slightly increased to 88%. If only cats without neurological signs were evaluated, sensitivity was 91% and specificity only 50% [130].

### 5.4. Aqueous Humor

In cats without FIP that do not present with effusion, uveitis is common and ocular signs (with or without involvement of the central nervous system) can be noticed in 60% of the affected cats [12,131]. Secondary to the inflammation of ocular structures and breakdown of the blood-ocular barrier, FCoV-bearing macrophages are present in the eye and FCoV antigen can be detected immunocytochemically within macrophages in the aqueous humor [47,132]. The results of the only prospective study that evaluated ICC in aqueous humor in cats with immunohistochemically confirmed FIP and control cats with similar clinical signs but histopathologically diagnosed other diseases are depicted in Table 8 [132]. The technique only has a moderate sensitivity and specificity for the diagnosis of FIP, and therefore, it cannot be used as a single confirming diagnostic test.

## 6. Detection of FCoV RNA by Reverse Transcriptase Polymerase Chain Reaction (RT-PCR)

Since its first application for the detection of FCoV [133], RT-PCR is frequently used to amplify FCoV RNA in different materials and to diagnose FIP. According to the long-existing hypothesis that only FIPV but not FECV is able to spread systemically, it was initially believed that detection of FCoV RNA via RT-PCR in blood, effusion or any other body fluid or tissue except for the gastrointestinal tract indicates presence of the virulent pathotype FIPV [6,101,104,134]. This hypothesis has, however, been refuted by several studies (Table 5, Table 6, Table 7, Table 8 and Table 9) and it is important to keep in mind that FCoV RNA can also be amplified outside of the gastrointestinal tract in cats without FIP. Nevertheless, it is well known that cats with FIP exhibit much higher viral loads than healthy FECV-infected cats [54,135,136,137]. Therefore, an ideal RT-PCR assay should be able to quantify the viral load in order to facilitate diagnosis. Not only are tissue samples from cats with FIP more likely to be FCoV-positive, but also viral loads are significantly higher in cats with FIP compared to healthy FECV-infected cats [135,137]. As an example, in a study analyzing a large number of tissue and fluid samples, 90% of the tissue samples and 78% of the fluid samples from cats with FIP were positive, whereas only 8% of the tissue samples and 2% of the fluid samples from control cats were positive for FCoV RNA. Additionally, relative FCoV copy number (a Ct value of 40 in RT-PCR was assigned to a relative copy number of 1, and a relative copy number was then calculated for each RT-PCR-positive sample using the following equation: 1.96^(40-Ct value)^) was significantly higher in tissue samples from cats with FIP than from FCoV-infected cats with other diseases or healthy FCoV-infected cats (median 8.3 × 10^3^ vs. median 25) [135]. Thus, a positive RT-PCR result with a high viral load is at least very suggestive for FIP [138].

### 6.1. Tissue

In cats with FIP, large quantities of FCoV RNA can only be found in tissues with inflammatory changes. Tissues which are not involved in the disease process contain only small amounts or no viral RNA at all. Among the organs with highest viral loads are the omentum, mesenteric lymph nodes and spleen, so these tissues are most useful for analysis by RT-PCR. The kidneys, liver, lung, myocardium and popliteal lymph nodes, in contrast, contain little or no viral RNA [57]. Studies using RT-PCR to target FCoV RNA in tissues are summarized in Table 9. However, in veterinary practice, RT-PCR is rarely applied to tissue samples to diagnose FIP, as this would usually require invasive tissue sample collection via laparotomy or laparoscopy or has to be done post mortem. Therefore, the studies cited here were mainly performed for research purposes and did not have the aim of evaluating RT-PCR in tissue samples as a diagnostic test for FIP.

When an early study applied RT-PCR to tissue samples (liver, kidney and/or spleen), FCoV RNA was identified in 88% cats with clinically suspected FIP; 100% of experimentally infected cats tested positive for FCoV by RT-PCR. However, FCoV RNA was also detected in the tissues from 61% of cats without FIP [133], indicating that the detection of FCoV RNA in tissue is not specific for FIP. Especially in hemolymphatic tissues (spleen, mesenteric lymph nodes, bone marrow), FCoV RNA could be detected in all cats with FIP but also in 85% of clinically healthy FECV-infected cats. In cats with FIP, real-time RT-PCR was positive in the spleen in 60% of cases, in mesenteric lymph nodes in 87% and in the bone marrow in 67% of cases. In healthy FECV-infected cats, FCoV RNA was found in the spleen in 38%, in mesenteric lymph nodes in 33% and in the bone marrow in 46% of cases [107]. As stated before, however, cats with FIP usually exhibit higher viral copy numbers in tissues than healthy FECV-infected cats [107,135,137].

In order to develop a RT-PCR assay which is more specific for FIP, one study evaluated a real-time RT-PCR detecting messenger RNA (mRNA) (and thus, replicating virus). It was hypothesized that this RT-PCR would only detect FIPV from cats with FIP but not FECV from healthy FCoV-infected cats. Indeed, in this study, tissues from healthy FECV-infected cats tested negative for FCoV mRNA in all cases [139], whereas FCoV mRNA was found in the tissues of 71% of cats with immunohistochemically confirmed FIP. Organs with the highest frequency of harboring FCoV mRNA in these cats were the mesenteric lymph nodes (100%), spleen (88%), lung (86%), liver (75%), bronchial lymph nodes (67%), kidneys (63%) and intestine (60%).

Recent studies evaluated the use of a quantitative RT-PCR (RT-qPCR) to detect FCoV RNA in FNA samples of the mesenteric lymph nodes and other abnormal tissues of clinical cases [119,140] and hypothesized that this technique would be a useful tool to diagnose FIP for veterinary practitioners, especially in cats without effusion. The first study included control cats that were either FCoV antibody-negative or antibody-positive but without signs indicative of FIP or with a confirmation of another cause of illness/death. Additionally, cats with non-effusive FIP, that was confirmed either by histopathology or a diagnostic algorithm, were included. In all of the cats, the mesenteric lymph nodes were sampled by minimally invasive ultrasound-guided FNA and examined by RT-qPCR. It was found that the detection of FCoV RNA in mesenteric lymph node FNA can be a sensitive and specific method to diagnose FIP (even when analyzed after shipping without any preservatives), although FCoV RNA was also detected in the mesenteric lymph nodes of a small proportion of cats without FIP, and therefore, was not completely specific for FIP [119]. The second study retrospectively included eleven cats with FIP without effusions and successfully amplified FCoV RNA from FNA samples of various organs in all of the cats. Since no control group was included in that study, specificity could not be evaluated [140]. The same finding was made in a study in which different samples (FNA or incisional biopsies (IB)) were obtained from different tissues (mesenteric and popliteal lymph nodes, liver, kidney, spleen, omentum) of 20 cats with immunohistochemically confirmed FIP in order to determine the prevalence of FCoV RNA in different tissues and to potentially evaluate the diagnostic performance of RT-PCR on those tissue samples for diagnosing FIP. Sensitivity was high in most tissues except for the popliteal lymph nodes and sensitivities did not greatly differ whether FNA or IB were used. Since no control group was included, specificity could not be determined [141].

### 6.2. Blood

Based on the erroneous assumption that only FIPV but not FECV is able to infect monocytes/macrophages and disseminate systemically, it was thought that the detection of FCoV RNA in blood was proof of an infection with FIPV, and thus, FIP. Therefore, RT-PCR was initially developed using primers for the highly conserved 3’-untranslated region (3’-UTR) of the FCoV genome in order to detect all known FCoV isolates. As a result, the RT-PCR could neither differentiate between FECV and FIPV, nor could it distinguish FCoV from CCV or TGEV. Nevertheless, as stated before, it was assumed that only FIPV would be detectable in blood. However, over the years, it has been shown that FCoV RNA could also be detected in the blood of asymptomatic cats and cats with diseases other than FIP [143,144]. Several studies evaluating the use of RT-PCR in serum, plasma or whole blood confirmed this finding of possible FCoV viremia in cats without FIP [16,80,106,145,146,147]. Additionally, a recent study demonstrated that FCoV viremia does not even predict the development of FIP, as none of the healthy cats that tested positive for genomic or replicating FCoV developed FIP or clinical illness within six months of testing [147].

All of the studies using RT-PCR on blood samples are summarized in Table 10. If different blood fractions were examined, sensitivity and specificity are presented as a range. Most studies used histopathology as reference standard [16,80,106,143,145,148,149]; one study also used the detection of FCoV antigen in macrophages in effusion [149]. Some studies had the limitation of including cats in which FIP was suspected clinically, but the diagnosis was not confirmed [148,150,151]. The control groups either consisted of healthy cats [106,143,145,147,148,150,151] and/or of cats with other diseases than FIP, sometimes showing clinical signs consistent with FIP [16,80,143,145,148,149].

One additional study worth mentioning is not shown in Table 10. This study included 63 serum samples from 62 cats with abdominal clinical signs. Since no definitive diagnosis was established in these cats, sensitivity and specificity could not be determined, and therefore, the study was not mentioned in Table 10. Nevertheless, it is worth noting that in this study, 18 cats had a positive result in a nested RT-PCR (RT-nPCR) assay. One of these cats survived for almost three years after examination; four additional cats lived for at least 70 months. Since median survival time for cats with FIP is very short (unless new antiviral drugs are applied) [152], it is likely that these cats did not have FIP and that the RT-nPCR was false positive [146].

Since FCoV viremia can be detected by RT-PCR in up to 80–90% of FECV-infected cats without FIP [106], detection of FCoV RNA in blood is not specific for FIP and RT-PCR in blood samples should not be used to confirm a diagnosis of FIP. Additionally, FCoV viral load is low in any blood fraction in experimental FIP [57] and this is most likely also the case in natural infection, leading to a rather low sensitivity of RT-PCR in blood. RT-PCR had better sensitivity when peripheral blood mononuclear cells (PBMC) were used compared to serum [149]; however, other studies came to differing results regarding sensitivity in the different blood fractions and most studies are not well comparable [80,106,141,143]. Sensitivity was poor in most of the studies; therefore, due to a rather low virus load and resulting low sensitivities, blood samples are not recommended for analysis by RT-PCR.

Although FECV can infect monocytes and macrophages, it is not capable of efficiently replicating within them [101,147,153,154]. In an attempt to increase specificity of RNA detection in blood, a RT-PCR specifically amplifying FCoV mRNA has been developed in order to detect only actively replicating virus in blood. In an initial study, this RT-PCR assay really did not detect FCoV mRNA in any of the control cats with clinical signs indicative of FIP but other confirmed diseases, whereas FCoV mRNA was found in 93% of cats with histopathologically confirmed FIP [148]. Nevertheless, subsequent studies also identified FCoV mRNA, and thus, replicating FCoV in 0.5–52% blood samples from healthy cats [147,148,150,151] and the detection of replicating virus did not predispose to the development of FIP [147]. Therefore, this special RT-PCR assay also is not adequately specific for FIP.

In general, however, cats with FIP exhibit higher viral loads and copy numbers than healthy FECV-infected cats [107,137] and higher viral copy numbers are found in the feces of healthy FECV-infected cats when compared to their blood [147]. Therefore, an RT-PCR that allows simultaneous amplification and quantification of viral mRNA could potentially distinguish FECV (with low viral copy numbers) from FIPV (with high viral copy numbers). A real-time RT-PCR based on primer-probe energy transfer detecting and quantifying subgenomic FCoV mRNA showed a very good sensitivity and specificity in one study, but was so far only applied on very small numbers of samples and not used in the field [139]. Loop-mediated isothermal amplification (LAMP) is a new molecular method with the advantage of delivering rapid in-house test results. Reverse transcriptase LAMP (RT-LAMP) has been used to amplify FCoV RNA in blood, effusion, lymph nodes and feces of cats with suspected FIP in a recent study, in which RT-nPCR was used as reference standard. Specificity of RT-LAMP was 100% in all materials; sensitivity, however, was only low to moderate [155].

### 6.3. Effusion

In cats with FIP that have effusion, virus load is much higher in the effusion than in blood [57]. Sensitivities and specificities of RT-PCR assays for the diagnosis of FIP in effusion are listed in Table 11. As shown, sample numbers are relatively low in some and especially in the older studies. Most studies used histopathology as reference standard [16,22,149,158,159]; in some studies, the detection of FCoV antigen in effusion [149,159] or tissue [159,160] or laboratory fluid analysis [158] was the reference standard. One study included 27 cats with a suspicion of FIP, of which only 13 had histopathologic confirmation of the disease and 8/13 cats had effusion [80]. Another study examined 61 effusion samples from cats with suspected FIP based on effusion feature criteria established by the European Advisory Board on Cat Diseases in 2009 [88]. The diagnosis was, however, not confirmed by reference standard methods in any of the cats [89]. The control groups consisted of cats with a clinical suspicion of FIP but different diagnoses [16,149,158,159,160].

One study examined a large number of effusion samples from 854 cats with clinically suspected FIP; the disease was, however, not confirmed. It could also not be determined whether cats with negative RT-PCR results had diseases other than FIP. Therefore, sensitivity and specificity could not be calculated, but FCoV RNA was detected in the effusion of 377/854 cats (44%) [161]. In another study, plasma and/or ascites from 42 cats with histopathologically confirmed FIP and 141 controls (healthy or with FIP-typical clinical signs but other diseases) were examined by RT-nPCR. FCoV RNA was detected in the plasma of 70% of cats with FIP. Five additional cats that tested negative in plasma had a positive RT-nPCR result in effusion. However, it was not stated how many effusion samples were analyzed in total [145]; thus, the study was not included in Table 11.

Although specificity of RT-PCR in effusion was high in many studies and a positive RT-PCR result can add to a suspicion of FIP, it has to be kept in mind that RT-PCR already detects small amounts of viral RNA, which can potentially leak from blood into effusion in the face of inflammation (even without FIP) in cats with circulating FECV [110]. Especially in recent studies, FCoV RNA could also be detected in the effusion of cats without FIP [15,135,157,159].

### 6.4. CSF

All studies evaluating RT-PCR for the detection of FCoV RNA in CSF have reported specificities of 100%, meaning that FCoV RNA could only be detected in the CSF of cats with FIP (with and without neurological signs) but not control cats (Table 12). Reference standards used in these studies was either histopathology [45,162] and/or the detection of FCoV antigen in effusion [162] or tissue [135]. The control groups consisted of cats with neurologic diseases only [45] or also included cats with non-neurologic diseases but clinical signs consistent with FIP [135,162].

Although the blood-brain barrier has not specifically been examined in cats with FIP yet, it can be assumed that it is impaired secondary to the inflammation caused by FIP [163]. In general, various infectious and inflammatory diseases of the central nervous system lead to the release of cytokines, adhesion molecules, metalloproteases and other mediators, which can damage tight junctions, basal membranes and cerebrovascular endothelium. This enables the leakage of cells and pathogens into the CSF across the blood-brain barrier [163]. Therefore, although false positive RT-PCR results have not been reported in CSF thus far, it seems theoretically possible that FECV could pass the blood-brain barrier in cats with neurologic diseases other than FIP, as it is also suspected for anti-FCoV antibodies [46]. One study detected a correlation between high anti-FCoV antibodies and positive RT-PCR in the CSF. In that study, FCoV RNA was found in only 15% of samples from cats with low, but in 100% of samples from cats with high CSF antibody titers. In contrast, FCoV RNA was not amplified in any of the antibody-negative cats. However, FIP was not confirmed in the cats in this study [90].

### 6.5. Aqueous Humor

Another type of sample material that can be examined for FCoV RNA by RT-PCR is the aqueous humor. To date, there have been very few studies that evaluated this sample material for analysis by RT-PCR in a small number of cats with FIP (Table 13). Additionally, a case report demonstrated positive RT-PCR in the aqueous humor of 3/10 cats with uveitis; however, there were no clear-defined FIP and control groups [131].

## 7. Detection of FCoV Mutations

FCoV replication, like replication of all RNA viruses, is prone to error [165]. Multiple individual mutations occur during each cycle of viral replication [12,58,166,167]. It is hypothesized that specific mutations or a combination of mutations then lead to the development of the virulent pathotype FIPV and trigger the tropism switch from enterocytes to macrophages as a key event in FIP pathogenesis [4,153,168].

Several different FCoV genes have been analyzed and suggested to harbor the mutation responsible for the FCoV pathotype switch. Open reading frame (ORF) 3abc, which is coding for the accessory proteins 3a, 3b and 3c, has been sequenced and compared in FCoV from cats with FIP and healthy cats and differences were detected. For instance, it was found that FIPV from tissue or effusion of cats with FIP contained a truncated protein 3c, whereas FECV from feces of healthy cats most often contained an intact protein 3c [108,167,169,170,171,172,173]. The 3a and 3b genes could play a role in FIP pathogenesis as well [174,175,176]. ORF 7ab, coding for accessory proteins 7a and 7b, has also been subject of studies looking into FIP pathogenesis, but somewhat contradictory results were found [77,153,169,170,176,177,178,179,180,181]. The FCoV spike (S) protein is responsible for viral cell entry. It includes a subunit for receptor binding (S1) and a subunit mediating membrane fusion (S2) [182,183,184]. Therefore, mutations in the FCoV S gene, more specific mutations in the S2 region and corresponding amino acid substitutions in the S protein, have been suggested to be responsible for the change of viral cell tropism [153,170,185,186,187]. As a result, only FIPV but not FECV are capable of efficient and sustained replication in macrophages, producing large amounts of viral particles and spreading the infection to adjacent cells [153,154]. Therefore, the S gene is of particular interest with regard to determining which mutations are responsible for the transition from FECV to FIPV. Recently, commercial diagnostic testing was introduced for FCoV S gene mutations.

When the S genes of a large number of FCoV derived from the feces of healthy cats and from tissues or ascites of cats with FIP were sequenced, two single nucleotide polymorphisms (SNP) were found that only were present in the FCoV from cats with FIP but not in the FCoV from healthy cats. These SNP were found at nucleotide position 23531 and 23537, which lie in close proximity within the S gene and the detection of one of the SNP allowed differentiation between FCoV from cats with FIP and healthy cats in 96% of the examined FCoV. In all of the sequenced fecal FCoV from healthy cats, adenine was found at nucleotide position 23531, whereas thymine or cytosine was detected in 92% of the FCoV from ascites or tissues of cats with FIP. Both SNP led to the substitution of methionine by leucine at amino acid position 1058 within the putative fusion peptide of the S protein (M1058L). In all of the sequenced fecal FCoV from healthy cats, thymine was detected at the second nucleotide position 23537, whereas guanine was found in 4% of the FCoV from ascites or tissues of cats with FIP. This SNP led to the substitution of serine to alanine at position 1060 of the S protein (S1060A) [185].

### 7.1. Tissue

After that first study provided evidence of an association of the two S gene mutations with FIP [185], a number of studies subsequently investigated the prevalence of the mutations in tissue samples from cats with and without FIP (Table 14).

Opinions vary substantially among researchers, especially on the specificity of S gene mutations for the FIP phenotype. For example, one recent study corroborated the initial findings by detecting M1058L in three tissue samples (mesentery, colonic lymph node and omentum) from cats with FIP; moreover, the mutation fully discriminated the FCoV found in these tissue samples from FCoV found in fecal samples from healthy cats [187]. In contrast, FCoV with substitution M1058L was not specific for the FIP phenotype in a different study, which also found the mutation in tissue samples from cats without FIP. Not only the majority of FCoV from tissue samples from cats with FIP (39/43; 91%), but also the majority of those from tissue samples from control cats without FIP (8/9; 89%) had a leucine (and thus, a mutated sequence) at position 1058 in that study. On the other hand, the majority of FCoV from fecal samples from cats with (10/13; 77%) and without FIP (6/6; 100%) had a methionine (and thus, a non-mutated sequence) at this position. Additionally, a number of tissue samples from cats with FIP (4/43; 9%) also exhibited a methionine at position 1058, which means that they did not contain a mutation despite being from cats with FIP. Therefore, the authors concluded that substitution M1058L was indicative of systemic spread of FCoV rather than a marker for FIP [137]. In this study, five tissue samples that did not contain the substitution M1058L were analyzed for substitution S1060A (the less common S gene mutation) by Sanger sequencing, and S1060A was then detected in 1/4 samples from cats with FIP but not in the one remaining sample from a control cat [137].

Since the first description, PCR assays detecting mutations M1058L and S1060A frequently have been used for diagnostic purposes and a number of studies have evaluated sensitivity and specificity or diagnostic accuracy of the detection of the mutations in the diagnosis of FIP (Table 14). However, largely contrasting results were also found in these studies, especially in terms of specificity, possibly as a result of different sequencing assays that were used.

One study, for example, examined a large number of tissue, fluid and fecal samples from cats with immunohistochemically confirmed FIP and controls in which FIP was excluded and performed RT-qPCR followed by pyrosequencing of an S gene amplicon in the RT-qPCR-positive samples. In total, 260 tissue samples from cats with FIP were analyzed. However, since histopathological data was only available for 225 of the tissue samples, only these numbers were included in the calculation of sensitivity and specificity (Table 14). Of these 225 samples, 202 were RT-qPCR-positive and S gene mutations were detected in 182/202 samples (M1058L being much more common than S1060A; mixed mutated and non-mutated FCoV or FCoV with both M1058L and S1060A also present). Of the 258 tissue samples from control cats, 19 were RT-qPCR-positive and 14/19 were positive for mutation M1058L. When comparing sensitivity and specificity of RT-qPCR alone with that of RT-qPCR plus S gene sequencing, specificity slightly increased from 93% to 95%, whereas sensitivity decreased from 90% to 81%. Therefore, it was concluded that the detection of FCoV S gene mutations did not improve the ability to diagnose FIP. Mutations were also found in cats without FIP, which supported the hypothesis of the mutations being a marker for systemic spread of FCoV and not for FIP [135]. A somewhat similar result was obtained a year later when a study compared different laboratory tests in tissue samples (mesenteric lymph nodes, spleen, small intestine, lung) from cats with FIP (positive IHC in at least one tissue) and control cats (histopathological diagnosis of another disease plus negative IHC). All samples were examined by RT-nPCR targeting the highly conserved 3’-UTR and sequencing was performed to detect S gene mutations. Again, the detection of FCoV S gene mutations improved specificity of the RT-nPCR from 50% to 88%, but sensitivity decreased from 91% to 70%, and an S gene mutation (type of mutation not shown) was also detected in a cat without FIP [15].

However, contrasting results were obtained when a real-time RT-PCR was developed and evaluated subsequently, which uses specific fluorescent hydrolysis probes to detect either one of the two SNP or the wildtype sequence. In that study, none of the 30 cats without FIP (with histopathological diagnosis plus negative IHC) tested positive for one of the S gene mutations in their tissues. Of the 34 cats with immunohistochemically confirmed FIP, 24 tested positive for one of the S gene mutations in at least one of their tissues (23/24 M1058L, one mixed genotype). In this study, sensitivity was 71% and as such comparable to the previous studies; however, specificity was excellent (100%) [142]. Finally, a recent study evaluated the same real-time RT-PCR detecting S gene mutations in 20 cats with immunohistochemically confirmed FIP. FNA and IB samples were obtained from the mesenteric and popliteal lymph nodes, spleen, liver, kidney and omentum and additionally from different body fluids. Samples were first examined by RT-PCR detecting the FCoV 7b gene (detecting all FCoV) and RT-PCR-positive samples were subsequently analyzed by real-time RT-PCR detecting S gene mutations. Interestingly, FCoV with S gene mutations was present in every cat, but the location of mutated FCoV varied from cat to cat. Sensitivity of the RT-PCR detecting all FCoV was good for all tissues except for the popliteal lymph nodes, but sensitivity decreased when S gene mutations were evaluated. Since sensitivity of FNA and IB did not significantly differ, sampling via minimally invasive FNA seems possible in order to obtain adequate samples for RT-PCR testing. Specificity was not determined in this study, since a control group was not included in the study protocol.

### 7.2. Blood

Diagnostic accuracy of the detection of FCoV S gene mutations has also been evaluated in blood samples (Table 15). RT-nPCR and subsequent S gene sequencing of serum and plasma samples from cats with FIP (diagnosed by histopathology ± IHC or by positive immunofluorescence in effusion) and control cats (diagnosed with another disease either ante or post mortem) revealed a sensitivity of only 7%, which confirms the very low virus load in blood. RT-nPCR was negative in all of the blood samples from cats without FIP, and thus, specificity of the sequencing step could not be determined [156]. Subsequent studies evaluated the aforementioned real-time RT-PCR detecting S gene mutations by specific fluorescent hydrolysis probes in blood samples (plasma, serum, buffy coat, whole blood) from cats with FIP [141] and/or controls [157] (reference standard histopathology and IHC) and either did not detect mutated FCoV in any of the blood samples or only found very low sensitivity. In some cats with FIP, FCoV RNA was detected, but viral concentrations were too low to allow pathotyping [157]. This implicates that blood cannot be recommended as sample material. Nevertheless, a study comparing different PCR tests including 3’-UTR RT-nPCR and a combined approach with RT-PCR followed by S gene sequencing reported a sensitivity of 75% for the RT-nPCR and 43% for combined RT-PCR and sequencing when whole blood was used. Potentially, whole blood can give better results than plasma or serum. Although the authors state that specificity of both RT-nPCR and combined approach were 100%, specificity of the sequencing step should not be calculated from that study, since none of the blood samples from cats without FIP tested positive for FCoV RNA at all [15].

### 7.3. Effusion

An association of substitutions M1058L and S1060A with FIP was shown by a study which detected amino acid substitution M1058L in 83% FCoV extracted from ascites of cats with FIP (reference standard for the diagnosis not reported) and a non-mutated sequence (a methionine codon) in 90% FCoV extracted from feces of healthy cats and 57% FCoV extracted from feces of cats with FIP [170]. However, contrasting results of PCR assays detecting FCoV S gene mutations were again, as in tissue and blood, found subsequently when analyzing effusion and other fluid samples for diagnostic purposes (Table 16).

One study including cats with FIP confirmed by IHC and control cats classified as non-FIP by confirmation of other diseases based on either histopathology and/or the definitive diagnosis of another disease ante mortem showed that RT-qPCR alone had a sensitivity of 85% and a specificity of 100%. Of the 17 RT-qPCR-positive cats, 11 had a mutated FCoV with substitution M1058L in their effusion detected by sequencing. One cat exhibited mutation S1060A. This resulted in a sensitivity of 60% for the detection of S gene mutations. Since none of the control cats were RT-qPCR positive, specificity of the sequencing step could not be determined. Thus, the majority of effusion samples that generated sequence data contained a mutated FCoV, although in that study population, detection of S gene mutations did not improve specificity [160]. When an RT-nPCR and subsequent S gene sequencing was used on effusion samples from cats with FIP (diagnosed by histopathology ± IHC or by positive immunofluorescence in effusion) and control cats (diagnosed with another disease either ante or post mortem), a similarly moderate sensitivity of 65% was detected. Of the 50 samples from cats with FIP, 36 were RT-nPCR-positive, and the majority of those (32/36) contained S gene mutations (M1058L *n* = 30, S1060A *n* = 2). RT-nPCR was negative in all of effusion samples from cats without FIP, and thus, specificity of the sequencing step again could not be determined [156]. Evaluations of a real-time RT-PCR detecting the S gene mutations with specific fluorescent hydrolysis probes in effusion from cats with FIP and controls (reference standard histopathology and IHC) detected similar sensitivities of 64–69% for the detection of S gene mutations [141,157]. Of the 35 samples from cats with FIP in one study, 34 were RT-PCR-positive. Mutated FCoV containing M1058L was detected in the majority of samples (22/34). Additionally, mixed FCoV (with and without S gene mutations) were detected in two cats. In 10 cats, RT-PCR was positive, but RT-PCR detecting S gene mutations was not successful, either because of a low virus load (*n* = 7) or despite a high virus load (*n* = 3). The reason for the latter could be the presence of unknown sequence variations or serotype II FCoV. RT-PCR was also positive in 3/24 effusion samples from cats without FIP and M1058L was detected in one ascites sample from a cat with chronic kidney disease [157]. Finally, one recent study collected different body fluids (CSF, aqueous humor, ascites, pleural or pericardial effusion) from cats with FIP (confirmed by IHC) and cats in which histopathological signs of FIP were absent. Of the 51 samples from cats with FIP, 40 were RT-qPCR-positive. In a subsequent pyrosequencing step, 30 of these 50 (one sample was lost from S gene analysis) contained mutated FCoV (M1058L *n* = 25, S1060A *n* = 1, mixed mutated and non-mutated FCoV without differentiation of mutation *n* = 4). Of the 47 fluid samples from cats without FIP, one was RT-qPCR-positive and contained substitution M1058L (an abdominal fluid sample from a cat with necrotizing pneumonia). Since individual results for effusion, CSF and aqueous humor are not shown in that study, sensitivity and specificity cannot be calculated individually, but only for all fluid samples together (Table 16). As was demonstrated for tissue samples, the detection of S gene mutations in this study led to a decrease in sensitivity (78% for RT-qPCR alone compared with 60% for combined approach) and an S gene mutation was also detected in a cat without FIP [135].

### 7.4. CSF

The detection of FCoV S gene mutations in CSF was evaluated in two studies presented as conference abstracts thus far (Table 17). One study obtained 16 CSF samples from cats with FIP (confirmed by IHC) and reported a sensitivity of 44%. Control cats were not included in that study [141]. The other study looked at 31 cats with immunohistochemically confirmed FIP (six with neurological signs) and 29 control cats with clinical signs indicative of FIP (10 with neurological signs) but definitively diagnosed other diseases, and calculated a sensitivity of 10%. Sensitivity increased to 17% if only cats with neurological signs were included. Since FCoV RNA was not detected in any of the control cats, specificity of the detection of S gene mutations could not be calculated [188].

### 7.5. Aqueous Humor

Only two studies that were presented as conference abstracts have evaluated sensitivity and specificity of the detection of FCoV S gene mutations in aqueous humor (Table 18). These studies included cats with FIP confirmed by IHC and reported sensitivities of only 10–17%. Specificity, however, could not be calculated in either one of the studies, either because no control cats were included or because none of the control cats were FCoV-positive at all [141,164].

## 8. Conclusions

**Figure 1:**
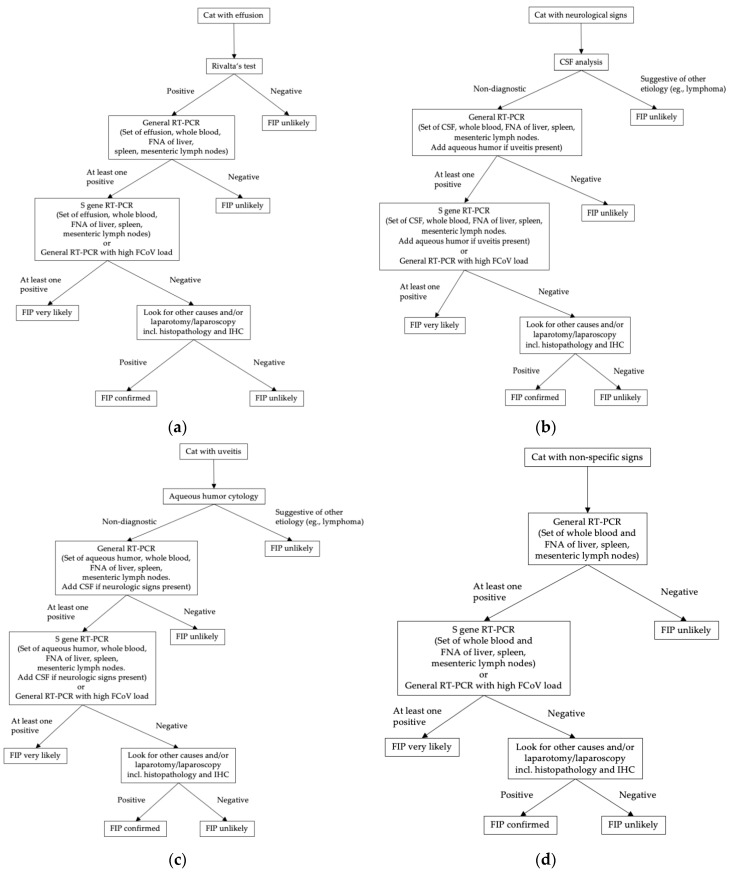
Decision trees depicting recommended diagnostic steps in a case suspicious of FIP, depending on a cat’s clinical presentation: (**a**) diagnostic steps recommended in a cat presenting with effusion; (**b**) diagnostic steps recommended in a cat presenting with neurological signs; (**c**) diagnostic steps recommended in a cat presenting with uveitis; (**d**) diagnostic steps recommended in a cat presenting with non-specific clinical signs. CSF = cerebrospinal fluid; FCoV = feline coronavirus; FIP = feline infectious peritonitis; FNA = fine-needle aspirates; IHC = immunohistochemistry; RT-PCR = reverse transcriptase polymerase chain reaction; S gene = spike gene.

Ante mortem diagnosis of FIP cannot be made based on results of one single diagnostic test. It is important to consider signalment, history, clinical signs and standard clinicopathological abnormalities in every cat that is presented with a suspicion of FIP. Additional tests, especially tests for direct virus detection, should be utilized depending on the clinical picture. Measurement of FCoV antibodies is not useful for the diagnosis at all. Routine hematology, serum biochemistry and, if present, analysis of effusion should be performed in every cat. In cats with neurological clinical signs, CSF analysis should be done as well. Diagnostic trees depicting relevant additional diagnostic steps that are recommended depending on a cat’s clinical presentation are shown in Figure 1. In cats with effusion, laboratory analysis of the fluid including Rivalta’s test should be performed. If this test is negative, FIP is rather unlikely, especially if the pre-test probability of FIP is low. If Rivalta’s test is positive, however, further diagnostic steps should follow. RT-PCR on effusion can help in establishing a diagnosis, especially if viral copy numbers are high. A negative RT-PCR on effusion makes FIP unlikely, unless suspicion is high based on clinical findings and other laboratory test results. High FCoV load or detection of S gene mutations in an RT-PCR-positive effusion sample can substantiate a suspicion of FIP. Additionally, in order to increase sensitivity of the detection of S gene mutations, other sample material, such as FNA of mesenteric lymph nodes, spleen and liver and whole blood can be included in the analysis. If, however, S gene mutations cannot be detected in an RT-PCR-positive sample and the virus load is low, but still the suspicion of FIP is high in a case, then more invasive diagnostic procedures, such as histopathology and IHC on tissue samples obtained in laparotomy/laparoscopy should be considered in order to confirm or exclude FIP.

In cats presenting without significant effusion, analysis of different sample types should be combined. Ultrasound-guided FNA of different organs including the mesenteric lymph nodes, spleen and liver can provide sample material for general RT-PCR, which should be the first diagnostic test in such a case. In cats with neurological clinical signs, diagnostic imaging and CSF sampling is an important step in reaching a diagnosis anyway and CSF should be submitted for RT-PCR as well. The same is true for cats with uveitis in which the aqueous humor should be integrated in a set of different samples for analysis by RT-PCR. By testing several materials, sensitivity of any given test can be increased. If general RT-PCR is positive in a cat without effusion, then again, the presence of FCoV S gene mutations or a high FCoV load might aid in cases of uncertainty—if mutations can be detected in an RT-PCR-positive sample, especially if copy numbers and pre-test probability of FIP are high, then FIP is likely. However, if S gene mutations cannot be detected in the set of samples or if general RT-PCR on the samples submitted is negative or virus load is low, then other causes for FIP should be ruled out as far as possible. If the suspicion of FIP is still high (based on signalment, clinical and other laboratory findings) in a case with negative RT-PCR or a case lacking S gene mutations, then histopathology and IHC on tissue samples must be considered in order to reach a definitive diagnosis.

However, as long as there is uncertainty regarding the putative role of S gene or other mutations in FIP pathogenesis, and since specificity of RT-PCR in any given sample is not absolute, an ideal diagnostic test for FIP still does not exist. IHC on histopathologically abnormal tissue obtained either post mortem or via laparotomy/laparoscopy still remains the gold standard of diagnosis.

## Figures and Tables

**Table 1:** Studies evaluating sensitivity and specificity of the detection of serum antibodies in comparison to either histopathology, a combination of diagnostic tests or clinical suspicion of feline infectious peritonitis (FIP). Control groups either consisted of healthy cats or cats with diseases other than FIP (with or without clinical signs consistent with FIP).

Study	Number of Samples	Antibody Assay	Sensitivity	Specificity	Reference Standard for FIP	Control Group
[19]	FIP (*n* = 39) Controls (*n* = 39)	IFAT	28–74%	64–92%	Histopathology	Diseases other than FIP
[78]	FIP (*n* = 70) Controls (*n* = 214)	IFAT	30–79%	64–98%	Histopathology	Diseases other than FIP
[16]	FIP (*n* = 97) Controls (*n* = 245)	IFAT	85%	57%	Histopathology	Diseases other than FIP
[87]	FIP (*n* = 88)	IFAT	100%	n. d.	Clinical suspicion	None
[79]	FIP (*n* = 19) Controls (*n* = 20)	Western blot	100%	25–45%	Combination of tests	Healthy or diseases other than FIP

IFAT = immunofluorescence antibody test; n. d. = not determined.

**Table 2:** Sensitivity and specificity from different studies evaluating the detection of antibodies in effusion for the diagnosis of feline infectious peritonitis (FIP) compared to histopathology, a combination of tests or a clinical suspicion of FIP. The control group of the first study consisted of cats with diseases other than FIP (with clinical signs consistent with FIP).

Study	Sample Type	Number of Samples	Antibody Assay	Sensitivity	Specificity	Reference Standard for FIP	Control Group
[16]	Ascites or pleural effusion	FIP (*n* = 119) Controls (*n* = 74)	IFAT	86%	85%	Histopathology	Diseases other than FIP
[87]	Ascites	FIP (*n* = 88)	IFAT	100%	n. d.	Clinical suspicion	None
[89]	Ascites or pleural effusion	FIP (*n* = 61)	IFAT	84%	n. d.	Combination of tests (according to ABCD guidelines)	None

ABCD = European Advisory Board on Cat Diseases; IFAT = immunofluorescence antibody test; n. d. = not determined.

**Table 3:** Sensitivity and specificity from different studies evaluating the detection of antibodies in cerebrospinal fluid (CSF) for the diagnosis of feline infectious peritonitis (FIP) compared to histopathology. Control groups consisted of cats with diseases other than FIP (with or without neurological signs).

Study	Number of Samples	Antibody Assay	Sensitivity	Specificity	Reference Standard for FIP	Control Group
[45]	FIP without neurological signs (*n* = 8)	IFAT	0%	n. d.	Histopathology	None
[45]	FIP with neurological signs (*n* = 16) Controls with neurological signs (*n* = 3)	IFAT	94%	100%	Histopathology	Neurologic diseases other than FIP
[46]	FIP without neurological signs (*n* = 13) Controls without neurological signs (*n* = 15)	IFAT	31%	100%	Histopathology	Diseases other than FIP
[46]	FIP with neurological signs (*n* = 10) Controls with neurological signs (*n* = 29)	IFAT	60%	93%	Histopathology	Diseases other than FIP
[90]	FIP with neurological signs (*n* = 271)	IFAT	10%	n. d.	Clinical suspicion	None

IFAT = immunofluorescence antibody test; n. d. = not determined.

**Table 4:** Sensitivity and specificity from a study evaluating the detection of immune complexes for the diagnosis of feline infectious peritonitis (FIP) compared to histopathology. The control group consisted of cats with diseases other than FIP (with clinical signs consistent with FIP).

Study	Sample Material	Number of Samples	Assay	Sensitivity	Specificity	Reference Standard for FIP	Control Cats
[16]	Serum	FIP (*n* = 29) Controls (*n* = 83)	Competitive ELISA	48%	91%	Histopathology	Diseases other than FIP

ELISA = enzyme-linked immunosorbent assay.

**Table 5:** Sensitivity and specificity from different studies evaluating immunostaining for the detection of feline coronavirus (FCoV) antigen in tissues samples. Feline infectious peritonitis (FIP) was confirmed either by histopathology alone or in combination with immunohistochemistry (IHC) of tissue samples. Control groups, if present, consisted of cats with diseases other than FIP with clinical signs consistent with FIP.

Study	Sample Material	Number of Samples	Assay	Sensitivity	Specificity	Reference Standard for FIP	Control Cats
[115]	Various tissues	FIP (*n* = 102) Controls (*n* = 6)	IHC	98%	100%	Histopathology	Diseases other than FIP
[117]	TCB liver	FIP (*n* = 25)	IHC	24%	n. d.	Histopathology/IHC of other organs	None
[117]	TCB kidney	FIP (*n* = 18)	IHC	31%	n. d.	Histopathology/IHC of other organs	None
[117]	FNA liver	FIP (*n* = 22)	ICC	17–31%	n. d.	Histopathology/IHC of other organs	None
[117]	FNA kidney	FIP (*n* = 24)	ICC	11–20%	n. d.	Histopathology/IHC of other organs	None
[116]	Various tissues	FIP (*n* = 26)	IHC	100%	n. d.	Histopathology	None
[118]	FNA mesenteric lymph nodes	FIP (*n* = 30) Controls (*n* = 11)	ICC	53%	91%	Histopathology/IHC of other organs	Diseases other than FIP

FNA = fine-needle aspirate; ICC = immunocytochemistry; n. d. = not determined; TCB = Tru-cut biopsies.

**Table 6:** Sensitivity and specificity from different studies evaluating immunostaining for the detection of feline coronavirus (FCoV) antigen in effusion samples. Feline infectious peritonitis (FIP) was confirmed either by histopathology alone or in combination with immunohistochemistry (IHC) of tissue samples. Control groups consisted of cats with diseases other than FIP with clinical signs consistent with FIP.

Study	Sample Material	Number of Samples	Assay	Sensitivity	Specificity	Reference Standard for FIP	Control Cats
[125]	Ascites or pleural effusion	FIP (*n* = 21) Controls (*n* = 11)	IFA	95%	100%	Histopathology	Diseases other than FIP
[78]	Ascites or pleural effusion	FIP (*n* = 49) Controls (*n* = 50)	IFA	69%	100%	Histopathology	Diseases other than FIP
[126]	Ascites or pleural effusion	FIP (*n* = 79) Controls (*n* = 31)	IFA	95%	100%	Histopathology/IHC	Diseases other than FIP
[16]	Ascites or pleural effusion	FIP (*n* = 109) Controls (*n* = 62)	IFA	57%	100%	Histopathology	Diseases other than FIP
[128]	Ascites or pleural effusion	FIP (*n* = 10) Controls (*n* = 7)	IFA	100%	71%	Histopathology	Diseases other than FIP
[127]	Ascites, pleural or pericardial effusion	FIP (*n* = 27) Controls (*n* = 29)	ICC	85%	72%	Histopathology/IHC	Diseases other than FIP

ICC = immunocytochemistry; IFA = immunofluorescence.

**Table 7:** Sensitivity and specificity from a study evaluating immunostaining for the detection of feline coronavirus (FCoV) antigen in cerebrospinal fluid (CSF) samples. Feline infectious peritonitis (FIP) was confirmed by histopathology and immunohistochemistry (IHC) of tissue samples. The control group consisted of cats with diseases other than FIP with clinical signs (neurological or non-neurological) consistent with FIP.

Study	Number of Samples	Assay	Sensitivity	Specificity	Reference Standard for FIP	Control Cats
[130]	FIP (*n* = 20) Controls (*n* = 18)	ICC	85%	83%	Histopathology/IHC	Diseases other than FIP
[130]	FIP with neurological signs (*n* = 9) Controls with neurological signs (*n* = 16)	ICC	78%	88%	Histopathology/IHC	Diseases other than FIP
[130]	FIP without neurological signs (*n* = 11) Controls without neurological signs (*n* = 2)	ICC	91%	50%	Histopathology/IHC	Diseases other than FIP

ICC = immunocytochemistry.

**Table 8:** Sensitivity and specificity from a study evaluating immunostaining for the detection of feline coronavirus (FCoV) antigen in aqueous humor samples. Feline infectious peritonitis (FIP) was confirmed by histopathology and immunohistochemistry (IHC) of tissue samples. The control group consisted of cats with diseases other than FIP with clinical signs (with or without uveitis) consistent with FIP.

Study	Number of Samples	Assay	Sensitivity	Specificity	Reference Standard for FIP	Control Cats
[132]	FIP (*n* = 26) Controls (*n* = 12)	ICC	64%	82%	Histopathology/IHC	Diseases other than FIP

ICC = immunocytochemistry.

**Table 9:** Sensitivity and specificity from different studies using reverse transcriptase polymerase chain reaction (RT-PCR) for the detection of feline coronavirus (FCoV) RNA in tissues from cats with feline infectious peritonitis (FIP) or control cats. FIP was confirmed by various methods and control groups either consisted of healthy cats or cats with diseases other than FIP (with or without clinical signs consistent with FIP).

Study	Sample Material	Number of Samples	RT-PCR Assay	Sensitivity	Specificity	Reference Standard for FIP	Control Cats
[133]	Liver, kidney, spleen	FIP (*n* = 8) Controls (*n* = 84)	RT-PCR	88%	39%	Histopathology	Diseases other than FIP
[133]	Liver, kidney, spleen	Experimentally induced FIP (*n* = 13)	RT-PCR	100%	n. d.	Experimen-tal infection	None
[107]	Hemolymphatic tissue	FIP (*n* = 15) Controls (*n* = 13)	Real-time RT-PCR	60–87%	54–67%	Histopathology/IHC	Healthy
[15]	Mesenteric lymph nodes, spleen, small intestine, lung	FIP (*n* = 11) Controls (*n* = 8)	RT-nPCR	91%	50%	Histopathology/IHC	Diseases other than FIP
[119]	FNA of mesenteric lymph nodes	FIP (*n* = 20) Controls (*n* = 26)	RT-qPCR	90%	96%	Histopathology or diagnostic algorithm	Healthy or diseases other than FIP
[139]	Various tissues	FIP (*n* = 32) Controls (*n* = 9)	Real-time RT-PCR for mRNA	60–100%	100%	Histopathology/IHC	Healthy
[137]	Various tissues	FIP (*n* = 45) Controls (*n* = 41)	Real-time RT-PCR	96%	78%	Histopathology/IHC	Diseases other than FIP
[140]	FNA of various tissues	FIP (*n* = 11)	RT-PCR	100%	n. d.	Histopathology	None
[135]	Various tissues	FIP (*n* = 57) Controls (*n* = 45)	RT-qPCR	98%	73%	Histopathology/IHC	Healthy or diseases other than FIP
[142]	Pooled tissues	FIP (*n* = 34) Controls (*n* = 30)	Real-time RT-PCR	94%	90%	Histopathology/IHC	Diseases other than FIP
[141]	Various tissues	FIP (*n* = 20)	Real-time RT-PCR	65–95%	n. d.	Histopathology/IHC	None

FNA = fine-needle aspirates; IHC = immunohistochemistry; mRNA = messenger RNA; n. d. = not determined; RT-nPCR = nested RT-PCR; RT-qPCR = quantitative RT-PCR.

**Table 10:** Sensitivity and specificity from different studies using reverse transcriptase polymerase chain reaction (RT-PCR) for the detection of feline coronavirus (FCoV) RNA in blood from cats with feline infectious peritonitis (FIP) or control cats. FIP was confirmed by various methods and control groups either consisted of healthy cats or cats with diseases other than FIP (with or without clinical signs consistent with FIP).

Study	Sample Material	Number of Samples	RT-PCR Assay	Sensitivity	Specificity	Reference Standard for FIP	Control Cats
[143]	Serum, plasma	FIP (*n* = 17) Controls (*n* = 15)	RT-nPCR	56–75%	75–88%	Histopathology	Healthy or diseases other than FIP
[145]	Plasma	FIP (*n* = 42) Controls (*n* = 141)	RT-nPCR	71%	89%	Histopathology	Healthy or diseases other than FIP
[106]	Serum, plasma, whole blood	FIP (*n* = 47) Controls (*n* = 69)	RT-snPCR	67–87%	10–20%	Histopathology	Healthy
[80]	Serum, plasma, whole blood	FIP (*n* = 6) Controls (*n* = 5)	RT-nPCR	20–75%	100%	Histopathology	Diseases other than FIP
[16]	Serum	FIP (*n* = 17) Controls (*n* = 8)	RT-nPCR	53%	88%	Histopathology	Diseases other than FIP
[148]	PBMC	FIP (*n* = 81) Controls (*n* = 17)	RT-PCR for mRNA	93%	100%	Histopathology	Healthy or diseases other than FIP
[148]	PBMC	FIP (*n* = 651) Controls (*n* = 424)	RT-PCR for mRNA	46%	95%	Histopathology or only clinical suspicion	Healthy or diseases other than FIP
[151]	Whole blood	FIP (*n* = 1) Controls (*n* = 25)	RT-PCR for mRNA	100%	48%	Clinical suspicion	Healthy
[150]	Whole blood	FIP (*n* = 10) Controls (*n* = 40)	RT-PCR	100%	33%	Clinical suspicion	Healthy
[150]	Whole blood	FIP (*n* = 10) Controls (*n* = 40)	RT-PCR for mRNA	100%	85%	Clinical suspicion	Healthy
[139]	WBC	FIP (*n* = 2)	Real-time RT-PCR for mRNA	100%	n. d.	Histopathology/IHC	Healthy
[149]	Serum, PBMC	FIP (*n* = 43) Controls (*n* = 49)	Real-time RT-PCR	15–29%	100%	Histopathology or detection of FCoV antigen in effusion	Diseases other than FIP
[155]	Whole blood	FCoV-positive(*n* = 4) FCoV-negative (*n* = 5)	RT-LAMP	25–50%	100%	RT-nPCR	Diseases other than FIP
[156]	Serum/plasma	FIP (*n* = 32) Controls (*n* = 21)	RT-nPCR	9%	100%	Histopathology, IHC or detection of FCoV antigen in effusion	Diseases other than FIP
[157]	Serum/plasma	FIP (*n* = 14) Controls (*n* = 3)	Real-time RT-PCR	14%	100%	Histopathology or IHC	Diseases other than FIP
[15]	Whole blood	FIP (*n* = 8) Controls (*n* = 8)	RT-nPCR	75%	100%	Histopathology/IHC	Diseases other than FIP
[147]	Buffy coat	Controls (*n* = 205)	RT-qPCR	n. d.	96%	None	Healthy
[147]	Buffy coat	Controls (*n* = 205)	RT-qPCR for mRNA	n. d.	99.5%	None	Healthy
[141]	Buffy coat, serum or whole blood	FIP (*n* = 20)	Real-time RT-PCR	36–77%	n. d.	Histopathology/IHC	None

IHC = immunohistochemistry; mRNA = messenger RNA; n. d. = not determined; PBMC = peripheral blood mononuclear cells; RT-LAMP = reverse transcriptase loop-mediated isothermal amplification; RT-nPCR = nested RT-PCR; RT-snPCR = semi-nested RT-PCR; RT-qPCR = quantitative RT-PCR; WBC = white blood cells.

**Table 11:** Sensitivity and specificity from different studies using reverse transcriptase polymerase chain reaction (RT-PCR) for the detection of feline coronavirus (FCoV) RNA in effusion from cats with feline infectious peritonitis (FIP) or control cats. FIP was confirmed by various methods and control groups either consisted of healthy cats or cats with diseases other than FIP (with or without clinical signs consistent with FIP).

Study	Sample Material	Number of Samples	RT-PCR Assay	Sensitivity	Specificity	Reference Standard for FIP	Control Cats
[143]	Ascites	FIP (*n* = 5) Controls (*n* = 1)	RT-nPCR	100%	100%	Histopathology	Healthy or diseases other than FIP
[158]	Ascites or pleural effusion	FIP (*n* = 12) Controls (*n* = 11)	RT-nPCR	91%	94%	Histopathology or laboratory fluid analysis	Diseases other than FIP
[80]	Ascites or pleural effusion	FIP (*n* = 5) Controls (*n* = 3)	RT-nPCR	100%	100%	Histopathology (some cats)	Diseases other than FIP
[16]	Ascites or pleural effusion	FIP (*n* = 5) Controls (*n* = 1)	RT-nPCR	100%	100%	Histopathology	Diseases other than FIP
[22]	Ascites or pleural effusion	FIP (*n* = 27)	RT-nPCR	96%	n. d.	Histopathology	None
[160]	Ascites, pleural or pericardial effusion	FIP (n = 20) Controls (*n* = 23)	Real-time RT-PCR	85%	100%	Histopathology/IHC	Diseases other than FIP
[149]	Ascites or pleural effusion	FIP (*n* = 36) Controls (*n* = 33)	Real-time RT-PCR	89%	100%	Histopathology or detection of FCoV antigen in effusion	Diseases other than FIP
[155]	Effusion	FCoV-positive (*n* = 5) FCoV-negative (*n* = 3)	RT-LAMP	40%	100%	RT-nPCR	Diseases other than FIP
[159]	Ascites or pleural effusion	FIP (*n* = 34) Controls (*n* = 37)	RT-LAMP	35–59%	95–97%	Histopathology or detection of FCoV antigen in effusion or tissue	Diseases other than FIP
[89]	Ascites or pleural effusion	FIP (*n* = 61)	RT-qPCR	85%	n. d.	Combination of tests (according to ABCD guidelines)	None
[135]	Ascites, pleural or pericardial effusion	FIP (*n* = 35) Controls (*n* = 28)	RT-qPCR	91%	96%	Histopathology/IHC	Healthy or diseases other than FIP
[156]	Ascites, pleural or pericardial effusion	FIP (*n* = 50) Controls (*n* = 51)	RT-nPCR	72%	100%	Histopathology, IHC or detection of FCoV antigen in effusion	Diseases other than FIP
[157]	Ascites or pleural effusion	FIP (*n* = 35) Controls (*n* = 24)	Real-time RT-PCR	97%	88%	Histopathology and/or IHC	Diseases other than FIP
[15]	Effusion	FIP (*n* = 10) Controls (*n* = 6)	RT-nPCR	100%	83%	Histopathology/IHC	Diseases other than FIP
[141]	Effusion	FIP (*n* = 14)	Real-time RT-PCR	86%	n. d.	Histopathology/IHC	None

ABCD = European Advisory Board on Cat Diseases; IHC = immunohistochemistry; n. d. = not determined; RT-LAMP = reverse transcriptase loop-mediated isothermal amplification; RT-nPCR = nested RT-PCR; RT-qPCR = quantitative RT-PCR.

**Table 12:** Sensitivity and specificity from different studies using reverse transcriptase polymerase chain reaction (RT-PCR) for the detection of feline coronavirus (FCoV) RNA in cerebrospinal fluid (CSF) from cats with feline infectious peritonitis (FIP) or control cats. FIP was confirmed by histopathology or detection of FCoV antigen and control groups consisted of cats with diseases other than FIP (either only neurologic or also non-neurologic diseases with clinical signs consistent with FIP).

Study	Sample Material	Number of Samples	RT-PCR Assay	Sensitivity	Specificity	Reference Standard for FIP	Control Cats
[45]	CSF	FIP (*n* = 24) Controls (*n* = 3)	RT-PCR	21%	100%	Histopathology	Neurologic diseases other than FIP
[45]	CSF	FIP with neurological signs (*n* = 16) Controls with neurological signs (*n* = 3)	RT-PCR	31%	100%	Histopathology	Neurologic diseases other than FIP
[162]	CSF	FIP (*n* = 19) Controls (*n* = 15)	Real-time RT-PCR	42%	100%	Histopathology or detection of FCoV antigen in effusion	Diseases other than FIP
[162]	CSF	FIP with neurological and/or ocular signs (*n* = 7) Controls with neurological and/or ocular signs (*n* = 3)	Real-time RT-PCR	86%	100%	Histopathology or detection of FCoV antigen in effusion	Diseases other than FIP
[135]	CSF	FIP (*n* = 14) Controls (*n* = 19)	RT-qPCR	50%	100%	Histopathology/IHC	Healthy or diseases other than FIP
[141]	CSF	FIP (*n* = 16)	Real-time RT-PCR	63%	n. d.	Histopathology/IHC	None

IHC = immunohistochemistry; n. d. = not determined; RT-qPCR = quantitative RT-PCR.

**Table 13:** Sensitivity and specificity from studies using reverse transcriptase polymerase chain reaction (RT-PCR) for the detection of feline coronavirus (FCoV) RNA in aqueous humor samples from cats with feline infectious peritonitis (FIP) or control cats. FIP was confirmed by immunohistochemistry (IHC) and control groups either consisted of cats with diseases other than FIP or cats euthanized due to behavioral conditions.

Study	Sample Material	Number of Samples	Assay	Sensitivity	Specificity	Reference Standard for FIP	Control Cats
[135]	Aqueous humor	FIP (*n* = 2)	RT-qPCR	50%	n. d.	IHC	Healthy or diseases other than FIP
[141]	Aqueous humor	FIP (*n* = 20)	Real-time RT-PCR	25%	n. d.	IHC	None
[164]	Aqueous humor	FIP (*n* = 25) Controls (*n* = 11)	Real-time RT-PCR	40%	100%	IHC	Diseases other than FIP

n. d. = not determined; RT-qPCR = quantitative RT-PCR.

**Table 14:** Sensitivity and specificity from different studies evaluating the detection of feline coronavirus (FCoV) spike (S) gene mutations in tissue samples. Feline infectious peritonitis (FIP) was confirmed by histopathology alone or in combination with immunohistochemistry (IHC), control cats were either healthy cats or cats with diseases other than FIP (with or without clinical signs consistent with FIP).

Study	Sample Type	Number of Samples	Assay	Sensitivity	Specificity	Reference Standard for FIP	Control Cats
[185]	Tissue or ascites	FIP (*n* = 118)	RT-nPCR plus S gene sequencing	96%	n. d.	Histopathology	None
[137]	Tissue	FIP (*n* = 47) Controls (*n* = 10)	RT-qPCR plus pyrosequencing	91% (M1058L)	11%	Histopathology/IHC	Diseases other than FIP
[187]	Tissue	FIP (*n* = 3)	RT-qPCR plus sequencing	100%	n. d.	Histopathology/IHC	None
[140]	FNA of various tissues	FIP (*n* = 9)	RT-PCR plus sequencing	89%	n. d.	Histopathology	None
[135]	Tissue	FIP (*n* = 225) Controls (*n* = 258)	RT-qPCR plus pyrosequencing ± Sanger sequencing	81%	95%	Histopathology/IHC	Healthy or diseases other than FIP
[15]	Tissue	FIP (*n* = 10) Controls (*n* = 8)	RT-nPCR plus sequencing	70%	88%	Histopathology/IHC	Diseases other than FIP
[142]	Pooled tissues	FIP (*n* = 34) Controls (*n* = 30)	Real-time RT-PCR	71%	100%	Histopathology/IHC	Diseases other than FIP
[141]	FNA or IB of various tissues	FIP (*n* = 20)	Real-time RT-PCR	15–50%	n. d.	Histopathology/IHC	None

FNA = fine-needle aspirates; IB = incisional biopsies; M1058L = substitution of methionine to leucine at position 1058 of the FCoV S protein; n. d. = not determined; RT-PCR = reverse transcriptase polymerase chain reaction; RT-nPCR = nested RT-PCR; RT-qPCR = quantitative RT-PCR.

**Table 15:** Sensitivity and specificity from different studies evaluating the detection of feline coronavirus (FCoV) spike (S) gene mutations in blood samples. Feline infectious peritonitis (FIP) was confirmed by histopathology alone, in combination with immunohistochemistry (IHC) or by positive immunostaining of FCoV antigen in effusion. Control cats were cats with diseases other than FIP (with clinical signs consistent with FIP).

Study	Sample Type	Number of Samples	Assay	Sensitivity	Specificity	Reference Standard for FIP	Control Cats
[156]	Serum/plasma	FIP (*n* = 31) Controls (*n* = 21)	RT-nPCR plus S gene sequencing	7%	n. d.	Histopathology, IHC or detection of FCoV antigen in effusion	Diseases other than FIP
[157]	Serum/plasma	FIP (*n* = 14) Controls (*n* = 3)	Real-time RT-PCR	0%	n. d.	Histopathology and/or IHC	Diseases other than FIP
[15]	Whole blood	FIP (*n* = 7) Controls (*n* = 7)	RT-nPCR plus S gene sequencing	43%	n. d.	Histopathology/IHC	Diseases other than FIP
[141]	Buffy coat, serum or whole blood	FIP (*n* = 20)	Real-time RT-PCR	0–23%	n. d.	Histopathology/IHC	None

n. d. = not determined; RT-PCR = reverse transcriptase polymerase chain reaction; RT-nPCR = nested RT-PCR.

**Table 16:** Sensitivity and specificity from different studies evaluating the detection of feline coronavirus (FCoV) spike (S) gene mutations in effusion samples. Feline infectious peritonitis (FIP) was confirmed by histopathology alone, in combination with immunohistochemistry (IHC) or by immunostaining of FCoV antigen in effusion. Control cats were either healthy cats or cats with diseases other than FIP (with or without clinical signs consistent with FIP).

Study	Sample Type	Number of Samples	Assay	Sensitivity	Specificity	Reference Standard for FIP	Control Cats
[170]	Ascites	FIP (*n* = 15)	RT-nPCR plus S gene sequencing	83%	n. d.	n. d.	None
[160]	Ascites, pleural or pericardial effusion	FIP (*n* = 20) Controls (*n* = 23)	RT-qPCR plus pyrosequencing	60%	n. d.	Histopathology/IHC	Diseases other than FIP
[135]	CSF, aqueous humor, ascites, pleural or pericardial effusion	FIP (*n* = 47) Controls (*n* = 50)	RT-qPCR plus pyrosequencing	60%	98%	Histopathology/IHC	Healthy or diseases other than FIP
[156]	Ascites, pleural or pericardial effusion	FIP (*n* = 49) Controls (*n* = 51)	RT-nPCR plus S gene sequencing	65%	n. d.	Histopathology, IHC or detection of FCoV antigen in effusion	Diseases other than FIP
[157]	Ascites or pleural effusion	FIP (*n* = 35) Controls (*n* = 24)	Real-time RT-PCR	69%	96%	Histopathology and/or IHC	Diseases other than FIP
[15]	Body cavity effusions	FIP (*n* = 10) Controls (*n* = 6)	RT-nPCR plus S gene sequencing	40%	83%	Histopathology/IHC	Diseases other than FIP
[141]	Body cavity effusions	FIP (*n* = 14)	Real-time RT-PCR	64%	n. d.	Histopathology/IHC	None

CSF = cerebrospinal fluid; n. d. = not determined; RT-PCR = reverse transcriptase polymerase chain reaction; RT-nPCR = nested RT-PCR; RT-qPCR = quantitative RT-PCR.

**Table 17:** Sensitivity and specificity from studies evaluating the detection of feline coronavirus (FCoV) spike (S) gene mutations in cerebrospinal fluid (CSF) samples. Feline infectious peritonitis (FIP) was confirmed by histopathology and immunohistochemistry (IHC). Control cats, if present, were cats with diseases other than FIP (with clinical signs consistent with FIP).

Study	Sample Type	Number of Samples	Assay	Sensitivity	Specificity	Reference Standard for FIP	Control Cats
[188]	CSF	FIP (*n* = 31) Controls (*n* = 29)	Real-time RT-PCR	10%	n. d.	Histopathology/IHC	Diseases other than FIP
[188]	CSF	FIP with neurological signs (*n* = 6) Controls with neurological signs (*n* = 10)	Real-time RT-PCR	17%	n. d.	Histopathology/IHC	Diseases other than FIP
[141]	CSF	FIP (*n* = 16)	Real-time RT-PCR	44%	n. d.	Histopathology/IHC	None

n. d. = not determined; RT-PCR = reverse transcriptase polymerase chain reaction.

**Table 18:** Sensitivity and specificity from different studies evaluating the detection of feline coronavirus (FCoV) spike (S) gene mutations in aqueous humor samples. Feline infectious peritonitis (FIP) was confirmed by histopathology and immunohistochemistry (IHC). Control cats, if present, were cats with diseases other than FIP (with clinical signs consistent with FIP).

Study	Sample Type	Number of Samples	Assay	Sensitivity	Specificity	Reference Standard for FIP	Control Cats
[141]	Aqueous humor	FIP (*n* = 20)	Real-time RT-PCR	10%	n. d.	Histopathology/IHC	None
[164]	Aqueous humor	FIP (*n* = 25) Controls (*n* = 11)	Real-time RT-PCR	17%	n. d.	Histopathology/IHC	Diseases other than FIP

n. d. = not determined; RT-PCR = reverse transcriptase polymerase chain reaction.
